# Microglial ERK-NRBP1-CREB-BDNF signaling in sustained antidepressant actions of (*R*)-ketamine

**DOI:** 10.1038/s41380-021-01377-7

**Published:** 2021-11-24

**Authors:** Wei Yao, Qianqian Cao, Shilin Luo, Lujuan He, Chun Yang, Jiaxu Chen, Qi Qi, Kenji Hashimoto, Ji-chun Zhang

**Affiliations:** 1 Guangzhou Key Laboratory of Formula-Pattern Research Center, School of Traditional Chinese Medicine, Jinan University, Guangzhou, China; 2 Department of Physiology, School of Medicine, Jinan University, Guangzhou, China; 3 Department of Pharmacy, The Second Xiangya Hospital, Central South University, Changsha, China; 4 Division of Clinical Neuroscience, Chiba University Center for Forensic Mental Health, Chiba, Japan; 5 MOE Key Laboratory of Tumor Molecular Biology, Clinical Translational Center for Targeted Drug, Department of Pharmacology, School of Medicine, Jinan University, Guangzhou, China; 6 Present Address: Department of Anesthesiology and Perioperative Medicine, The First Affiliated Hospital of Nanjing Medical University, Nanjing, China

**Keywords:** Neuroscience, Depression

## Abstract

(*R,S*)-ketamine elicits rapid-acting and sustained antidepressant actions in treatment-resistant patients with depression. (*R*)-ketamine produces longer-lasting antidepressant effects than (*S*)-ketamine in rodents; however, the precise molecular mechanisms underlying antidepressant actions of (*R*)-ketamine remain unknown. Using isobaric Tag for Relative and Absolute Quantification, we identified nuclear receptor-binding protein 1 (NRBP1) that could contribute to different antidepressant-like effects of the two enantiomers in chronic social defeat stress (CSDS) model. NRBP1 was localized in the microglia and neuron, not astrocyte, of mouse medial prefrontal cortex (mPFC). (*R*)-ketamine increased the expression of NRBP1, brain-derived neurotrophic factor (BDNF), and phosphorylated cAMP response element binding protein (p-CREB)/CREB ratio in primary microglia cultures thorough the extracellular signal-regulated kinase (ERK) activation. Furthermore, (*R*)-ketamine could activate BDNF transcription through activation of CREB as well as MeCP2 (methyl-CpG binding protein 2) suppression in microglia. Single intracerebroventricular (i.c.v.) injection of CREB-DNA/RNA heteroduplex oligonucleotides (CREB-HDO) or BDNF exon IV-HDO blocked the antidepressant-like effects of (*R*)-ketamine in CSDS susceptible mice. Moreover, microglial depletion by colony-stimulating factor 1 receptor (CSF1R) inhibitor PLX3397 blocked the antidepressant-like effects of (*R*)-ketamine in CSDS susceptible mice. In addition, inhibition of microglia by single i.c.v. injection of mannosylated clodronate liposomes (MCLs) significantly blocked the antidepressant-like effects of (*R*)-ketamine in CSDS susceptible mice. Finally, single i.c.v. injection of CREB-HDO, BDNF exon IV-HDO or MCLs blocked the beneficial effects of (*R*)-ketamine on the reduced dendritic spine density in the mPFC of CSDS susceptible mice. These data suggest a novel ERK-NRBP1-CREB-BDNF pathways in microglia underlying antidepressant-like effects of (*R*)-ketamine.

## Introduction

Depression is the most common psychiatric disorder in the world. The currently available antidepressants have important limitations such as delayed onset from weeks to months and low rates of efficacy. The serendipitous discovery of the robust antidepressant actions of the *N*-methyl-D-aspartate receptor (NMDAR) antagonist (*R,S*)-ketamine in depressed patients has led to new avenues in mood disorders [1–4]. In 2000, Berman et al. [5] reported rapid-acting and sustained antidepressant effects of (*R,S*)-ketamine in patients with depression. Subsequently, several groups replicated the robust antidepressant effects of (*R,S*)-ketamine in treatment-resistant patients with major depression or bipolar disorder [6–12]. Furthermore, (*R,S*)-ketamine rapidly reduced suicidal ideation in patients with severe depression [13, 14]. Meta-analyses revealed that (*R,S*)-ketamine has rapid-acting and sustained antidepressant effects, and anti-suicidal ideation effects in treatment-resistant patients with depression [15–18]. Importantly, meta-analyses showed that the effect sizes of (*R,S*)-ketamine are larger than those of other NMDAR antagonists [15, 16], suggesting that NMDAR does not play a major role in the robust antidepressant effects of (*R,S*)-ketamine. However, the precise molecular and cellular mechanisms underlying antidepressant effects of (*R,S*)-ketamine remain poorly understood [19–24].

(*R,S*)-Ketamine (Ki = 0.53 μM for NMDAR) is a racemic mixture that contains equal amounts of (*R*)-ketamine (or arketamine) (Ki = 1.4 μM for NMDAR) and (*S*)-ketamine (or esketamine) (Ki = 0.30 μM for NMDAR) [25]. Increasing preclinical data show that (*R*)-ketamine displays greater potency and longer-lasting antidepressant-like effects than (*S*)-ketamine in rodent models of depression [26–31]. In both rodents and monkey, the side-effects of (*R*)-ketamine were lower than those of (*R,S*)-ketamine and (*S*)-ketamine [27, 32–36]. In humans, the incidence of side-effects (i.e., psychotomimetic and dissociative effects) of (*R*)-ketamine was lower than that of (*S*)-ketamine [37–39]. It is recognized that (*S*)-ketamine contributes to the acute side-effects of (*R,S*)-ketamine, whereas (*R*)-ketamine may not be associated with these side-effects [20]. Taken together, it is likely that (*R*)-ketamine could be a new antidepressant without side-effects of (*R,S*)-ketamine [2, 22–24, 40].

Mounting evidence suggests a key role of brain-derived neurotrophic factor (BDNF) in antidepressant-like effects of (*R,S*)-ketamine and its two enantiomers in rodents [2, 22–24, 41–44]. Shirayama et al. [45] reported that direct injection of BDNF in the hippocampus caused long-lasting (i.e., 10 days) antidepressant-like effects in rat learned helplessness model, suggesting a role of BDNF in the long-lasting antidepressant effects. However, the precise molecular mechanisms underlying the relationship between (*R*)-ketamine’s long-lasting antidepressant actions and BDNF signaling remain poorly understood.

The isobaric Tags for Relative and Absolute Quantification (iTRAQ)-based proteomic technique has been widely used in proteomic workflows relative quantification [46]. The aim of this study was to identify the novel molecular mechanisms underlying long-lasting antidepressant-like effects of (*R*)-ketamine in rodents. Here, we conducted iTRAQ analysis of the medial prefrontal cortex (mPFC) of chronic social defeat stress (CSDS) susceptible mice treated with either (*R*)-ketamine or (*S*)-ketamine since mPFC is implicated in the antidepressant-like effects of (*R,S*)-ketamine and two enantiomers [27, 47, 48]. Here, we identified the nuclear receptor-binding protein 1 (NRBP1) as differentially expressed protein for two enantiomers. Furthermore, we investigated the role of NRBP1, upstream and downstream signaling such as the extracellular signal-regulated kinase (ERK), cAMP response element binding protein (CREB), and BDNF in the antidepressant-like effects of (*R*)-ketamine.

## Methods and materials

### Animals

Detailed information of animals was shown in the Supplementary Information.

### Chronic social defeat stress (CSDS) model

CSDS was performed according to the previous reports [49–53] (for details, see Supplementary Information).

### Compounds and cell cultures

Detailed information of the compounds including (*R*)-ketamine, (*S*)-ketamine, (2*R*,6*R*)-hydroxynorketamine [(2*R*,6*R*)-HNK], lipopolysaccharide (LPS), SL327 (ERA inhibitor), PLX3397 [colony-stimulating factor 1 receptor (CSF1R) inhibitor], mannosylated clodronate liposomes (MCLs), the antisense oligonucleotides and cRNA for targeting CREB or BDNF exon IV, and CREB-DNA/RNA heteroduplex oligonucleotides (HDO) or BDNF exon IV-HDO was shown in the Supplementary Information. Detailed information of cells cultures such as HEK293T, BV2 cells and primary microglia was also shown in the Supplementary Information.

### iTRAQ analysis

(*R*)-ketamine (10 mg/kg as HCL salt) or (*S*)-ketamine (10 mg/kg as HCL salt) was administered intraperitoneally (i.p.) to CSDS susceptible mice. Tissues of mPFC were collected 7 days after a single administration of ketamine enantiomers. iTRAQ analysis of mPFC samples was performed at Aproscience Co., Ltd (now: Integrale Co., Ltd., Naruto, Tokushima, Japan).

### Injection and behavioral tests

Detailed method of intracerebroventricular (i.c.v.) injection was shown in Supplementary Information. (*R*)-ketamine (10 mg/kg as HCL salt) was administered i.p. to CSDS susceptible mice. Behavioral tests, including locomotion test (LMT), forced swimming test (FST), and 1% sucrose preference test (SPT), were performed according to the previous reports [50–54] (for details, see Supplementary Information). Behavioral experiments were performed in a blind manner.

### Immunoprecipitation, quantitative real-time PCR assay, western blotting assay, luciferase assay, chromatin immunoprecipitation (ChIP) assay, immunofluorescence staining, and dendritic spine analysis

We performed immunoprecipitation, quantitative real-time PCR, western blot, luciferase assay, ChIP assay, immunofluorescence staining, and dendritic spine analysis for in vitro and/or in vivo experiments (for details, see Supplementary Information).

### Statistical analysis

All data results were expressed as the mean ± standard error of the mean (S.E.M.). All data were analyzed using PASW Statistics 20 software. Differences among the groups were evaluated using one-way analysis of variance, followed by post hoc Fisher least significant difference test. Student’s *t* test was used to compare the differences between two groups. The *P* values <0.05 were considered to be significant.

## Results

### (*R*)-ketamine induces CREB activation through NRBP1 and ERK

To investigate the molecular mechanism of long-lasting antidepressant-like effects of (*R*)-ketamine, we performed iTRAQ analysis to discover the different expressions of proteins in the mPFC of CSDS susceptible mice 7 days after (*R*)-ketamine (10 mg/kg) or (*S*)-ketamine (10 mg/kg) administration. The nuclear receptor-binding protein 1 (NRBP1) was the most differentially expressed protein between the two enantiomers (Table S1). NRBP1 is an adapter protein that is ubiquitously expressed across all cell types, and it plays a role in cellular homeostasis [55, 56]. First, we examined the cellular expression of NRBP1 in the mPFC of adult mouse brain. Immunoreactivity for NRBP1 was expressed in the neurons (for Camk2α^+^ and GABA^+^) and microglia (for CD11b^+^), but not astrocyte (for GFAP) of mPFC (Fig. S1).

A computer-assisted generation of a protein-interaction database suggests that NRBP might bind to CREB [57]. The immunoprecipitation assay showed that NRBP1 and CREB bind to each other under physiological function and after (*R*)-ketamine (10 μM) or (*S*)-ketamine (10 μM) treatment (Fig. S2). However, the precise physiological function of the interaction of NRBP1 and CREB is unclear. We previously reported that ERK plays a role in the antidepressant-like effects of (*R*)-ketamine in CSDS model [30]. It is also known that phosphorylation of ERK could activate the transcription factor CREB, resulting in the regulation of BDNF transcription [58, 59]. Together, we have hypothesis that (*R*)-ketamine may activate the expression of BDNF though NRBP1 and ERK-CREB signaling. To address the hypothesis, we examined the relationship between NRBP1 and ERK-CREB. Western blot assay showed that siRNA-NRBP1 caused down-regulation of NRBP1 and the ratio of p-CREB/CREB in the BV2 cells, in a concentration dependent manner (Fig. S3A). Next, we examined the effects of ketamine enantiomers on the expression of NRBP1 and p-CREB/CREB ratio in the primary microglia. (*R*)-ketamine significantly increased the expression of NRBP1 and p-CREB/CREB ratio in the primary microglia, in a concentration dependent manner (Fig. S3B). In contrast, (*S*)-ketamine increased expression of NRBP1 at 1 μM and p-CREB/CREB ratio at 10 μM (Fig. S3B). (*R*)-ketamine was more potent than (*S*)-ketamine (Fig. S3B). To examine the role of ERK, we treated differential concentrations of ERK inhibitor SL327 for primary microglia. Western blot analysis showed that increased expressions of NRBP1, BDNF, ratio of p-ERK/ERK, and p-CREB/CREB by (*R*)-ketamine were significantly attenuated by SL327 (Fig. S3C). The results suggest that (*R*)-ketamine activates phosphorylation of CREB though activation of NRBP1 and ERK, resulting in BDNF upregulation.

### Effects of two ketamine enantiomers on BDNF transcription

**Fig. 1:**
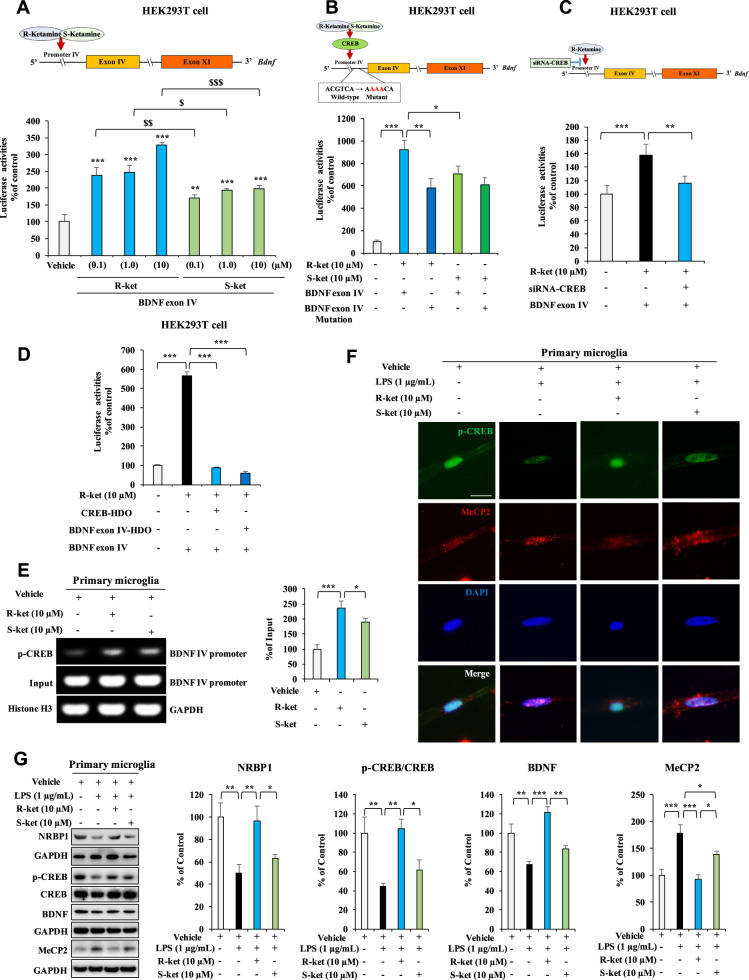
Effects of (*R*)-ketamine and (*S*)-ketamine on the BDNF activation. **A**, **B** The luciferase assay for BDNF exon IV promoter. **A** BDNF exon IV promoter activity in the HEK293T cells treated with (*R*)-ketamine (0.1, 1.0, and 10 μM) or (*S*)-ketamine (0.1, 1.0, and 10 μM). The data are the mean ± SEM (*n* = 6). **P < 0.01; ***P < 0.001 compared to vehicle group. ^$^*P* < 0.05; ^$$^*P* < 0.01; ^$$$^*P* < 0.001 (one-way ANOVA). **B** BDNF exon IV promoter activity in the HEK293T cells treated with (*R*)-ketamine (10 μM) [or (*S*)-ketamine (10 μM)] with or without mutation (Mut) plasmids. The data are the mean ± SEM (*n* = 9 or 10). **P < 0.01; ***P < 0.001 (one-way ANOVA). **C** BDNF exon IV promoter activity in the HEK293T cells treated with (*R*)-ketamine (10 μM) with or without siRNA-CREB plasmids. The data are the mean ± SEM (*n* = 4). **P < 0.01; ***P < 0.001 (one-way ANOVA). **D** BDNF exon IV promoter activity in the HEK293T cells treated with (*R*)-ketamine (10 μM) with or without CREB-HDO or BDNF exon IV-HDO. The data are the mean ± SEM (*n* = 10). **P < 0.01; ***P < 0.001 (one-way ANOVA). **E** ChIP-PCR assay for BDNF IV promoter. The p-CREB protein–DNA crosslinking samples were obtained from the primary microglia treated with vehicle, (*R*)-ketamine (10 μM), or (*S*)-ketamine (10 μM) via co-immunoprecipitating with anti-p-CREB. PCR was carried out by using BDNF exon IV promoter primer. The data are the mean ± SEM (*n* = 4). *P < 0.05; ***P < 0.001 (one-way ANOVA). **F** The immunofluorescence for p-CREB and MeCP2 in the LPS (1 µg/mL)-treated primary microglia with (*R*)-ketamine (10 µM) or (*S*)-ketamine (10 µM). Scale bar = 50 μm. **G** Expression of NRBP1, BDNF, MeCP2, and the ratio of p-CREB/CREB in the LPS-treated primary microglia with (*R*)-ketamine (10 µM) or (*S*)-ketamine (10 µM). The data are the mean ± SEM (*n* = 5). *P < 0.05; **P < 0.01; ***P < 0.001 (one-way ANOVA).

It is reported that CREB functions as a transcription activator of BDNF via motif ahead of *Bdnf* exon IV [60, 61]. Here, we examined whether (*R*)-ketamine can promote BDNF expression by affecting *Bdnf* transcription through the activation of CREB. First, we analyzed the DNA sequences of the promoter regions in the mouse *Bdnf* exon IV by using luciferase assay. Both (*R*)-ketamine and (*S*)-ketamine could activate *Bdnf* exon IV promoter in HEK293T cells, in a concentration dependent manner (Fig. 1A). Interestingly, activation for *Bdnf* exon IV promoter by (*R*)-ketamine was more potent than (*S*)-ketamine (Fig. 1A). In contrast, (2*R*,6*R*)-HNK (the metabolite from (*R*)-ketamine) [28] did not activate *Bdnf* exon IV promoter whereas its parent compound (*R*)-ketamine significantly activated *Bdnf* exon IV promoter (Fig. S4). Furthermore, the mutation in this motif significantly attenuated the promoter activity by (*R*)-ketamine or (*S*)-ketamine (Fig. 1B). Moreover, activation of *Bdnf* exon IV promotor by (*R*)-ketamine was significantly blocked by siRNA-CREB, CREB-HDO, and BDNF exon IV-HDO (Fig. 1C, D).

The ChIP assay using specific p-CREB antibody showed that (*R*)-ketamine interacted with the *Bdnf* exon IV promoter, and that (*R*)-ketamine was more potent than (*S*)-ketamine (Fig. 1E). It is known that methyl-CpG-binding protein 2 (MeCP2) is a transcriptional regulator of BDNF [53, 62, 63]. Immunofluorescence staining revealed that LPS treatment caused the redistribution of p-CREB and MeCP2 for the nucleus of primary microglia (Fig. 1F) and BV2 cells (Fig. S4). More MeCP2 within the nucleus and the more diffused nuclear staining pattern of p-CREB in vehicle-treated cells became more punctate upon LPS treatment, and this phenomenon could be reversed by (*R*)-ketamine or (*S*)-ketamine (Figs. 1F, S5). Interestingly, (*R*)-ketamine was more potent than (*S*)-ketamine (Figs. 1F, S5).

Western blot analysis showed that (*R*)-ketamine, but not (*S*)-ketamine, significantly attenuated the decreased expressions of NRBP1, BDNF, and the reduced ratio of p-CREB/CREB in the LPS-treated primary microglia (Fig. 1G). In contrast, both (*R*)-ketamine and (*S*)-ketamine significantly attenuated the increased expression of MeCP2 in the LPS-treated primary microglia; however, (*R*)-ketamine was more potent than (*S*)-ketamine (Fig. 1G). In addition, siRNA-CREB significantly blocked amelioration of abnormal changes in p-CREB/CREB ratio, BDNF and MeCP2 expression in the LPS-treated BV2 cells by (*R*)-ketamine (Fig. S6). These results suggest that (*R*)-ketamine could activate BDNF transcription through the activation of CREB as well as MeCP2 suppression in microglia.

### Effects of (*R*)-ketamine on depression-like behaviors, alterations in the expressions of NRBP1, BDNF, MeCP2, and p-CREB/CREB ratio in CSDS susceptible mice

On day 12, vehicle (10 mg/kg) or (*R*)-ketamine (10 mg/kg) was administered i.p. to CSDS susceptible mice. LMT, FST, and SPT were performed on day 19, day 20, and day 21, respectively (Fig. S7A). There were no changes in locomotion among the three groups (Fig. S7B). (*R*)-ketamine significantly attenuated the increased immobility time of FST in the CSDS susceptible mice (Fig. S7C). In the SPT, (*R*)-ketamine significantly attenuated the decreased sucrose preference in the CSDS susceptible mice (Fig. S7D). The results suggest that (*R*)-ketamine shows long-lasting antidepressant-like effects in CSDS model, consistent with our previous reports [27, 30, 64, 65].

Expressions of NRBP1 and BDNF, the ratio of p-CREB/CREB in the mPFC from CSDS susceptible mice were significantly lower than those of control group although expression of MeCP2 in the CSDS susceptible mice was higher than control mice (Fig. S7E). Interestingly, (*R*)-ketamine significantly improved these changes in the mPFC of CSDS susceptible mice (Fig. S7E). Furthermore, immunofluorescence showed that (*R*)-ketamine significantly improved the reduced fluorescence intensity of p-CREB in the mPFC of CSDS susceptible mice (Fig. S7F).

### Effects of CREB-HDO on the antidepressant-like effects of (*R*)-ketamine

**Fig. 2:**
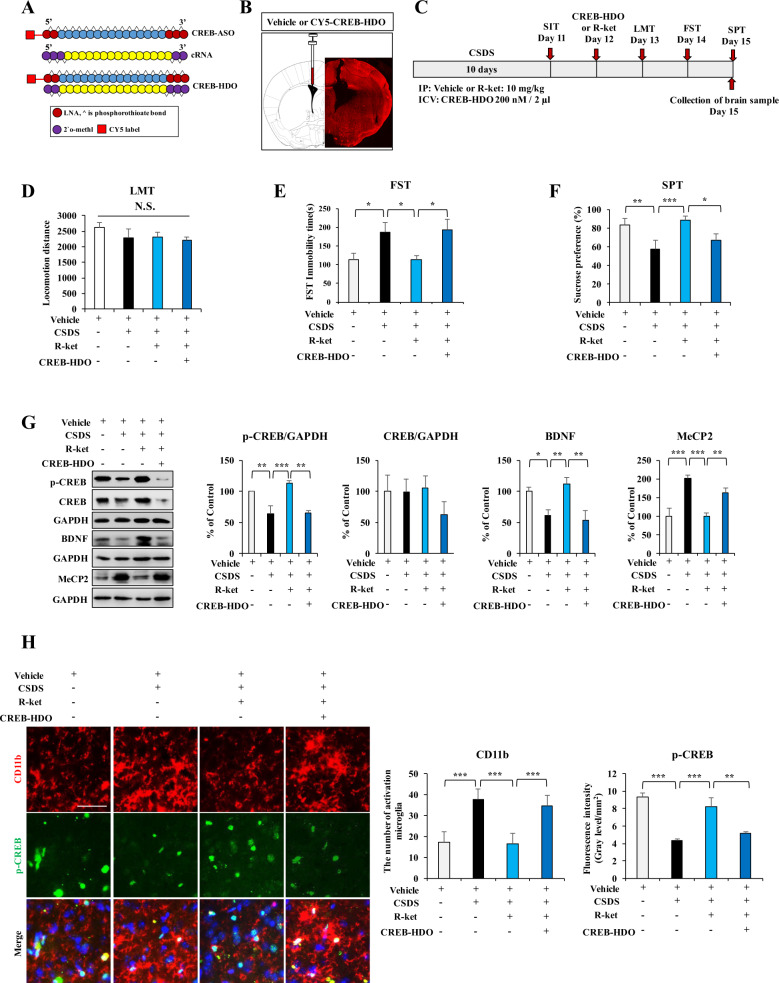
Effects of CREB-HDO on beneficial effects of (*R*)-ketamine on depression-like behaviors, and expression of p-CREB, CREB, BDNF, MeCP2, and CD11b in the mPFC. **A** Schematic illustration of the construction of ASO, cRAN, and HDO. **B** Graphical illustration of intracerebroventricular (i.c.v.) injection site and the distribution of CY5-CREB-HDO in the brain. **C** The schedule of CSDS, treatment, behavioral tests, and sample collection. CSDS was performed from day 1 to day 10. On day 11, social interaction test (SIT) was performed to select CSDS susceptible mice. CREB-HDO or vehicle was injected i.c.v. to mice 30 min before i.p. injection of (*R*)-ketamine (10 mg/kg). Subsequently, behavioral tests such as locomotion tests (LMT), forced swimming test (FST), and sucrose preference test (SPT) were performed. **D** LMT. **E** FST. **F** SPT. The data are the mean ± SEM (*n* = 7 or 8). *P < 0.05; **P < 0.01; ***P < 0.001 (one-way ANOVA). **G** The expression of p-CREB, CREB, BDNF, and MeCP2 in the mPFC. The data are the mean ± SEM (*n* = 5). *P < 0.05; **P < 0.01; ***P < 0.001 (one-way ANOVA). **H** The immunofluorescence analysis for p-CREB and CD11b in the mPFC. The data are the mean ± SEM (*n* = 5). **P < 0.01; ***P < 0.001 (one-way ANOVA). Scale bar = 50 μm.

For gene silencing, we designed CREB-HDO that carried locked nucleic acids at each end flanking the central base of DNA and with or without CY5 label, and carried 2′-O-methyl at each end flanking the central base of cRNA (Fig. 2A). CY5-CREB-HDO was widely distributed in the mouse brain 72 h by i.c.v. injection (Fig. 2B). Western blot assay showed that CREB-HDO significantly decreased the expression of CREB and BDNF in the BV2 cells (Fig. S8A). Furthermore, i.c.v. injection of CREB-HDO significantly decreased the expression of CREB and BDNF in the mPFC of adult mice (Fig. S8B). These results indicate that CREB-HDO can be efficiently distributed across the mouse brain and silenced *Creb* gene expression.

Single i.c.v. injection of CREB-HDO (200 nM, 2 μl) significantly attenuated the antidepressant-like effects of (*R*)-ketamine (Fig. 2C–F). There were no changes in locomotion among the four groups (Fig. 2D). In the FST and SPT, CREB-HDO significantly blocked antidepressant-like effects of (*R*)-ketamine in the CSDS susceptible mice (Fig. 2E, F).

Western blot assay showed that CREB-HDO significantly blocked the beneficial effects of (*R*)-ketamine on alterations in the expression of p-CREB, BDNF and MeCP2 in the mPFC of CSDS susceptible mice (Fig. 2G). Immunofluorescence staining showed that immunoreactivity of CD11b and p-CREB showed co-localization in the mPFC (Fig. 2H). Furthermore, CSDS susceptible mice showed increased CD11b immunoreactivity in the mPFC, whereas these mice showed decreased p-CREB immunoreactivity in the mPFC (Fig. 2H). Moreover, (*R*)-ketamine significantly improved both decreased CD11b immunoreactivity and increased p-CREB immunoreactivity in the mPFC of CSDS susceptible mice (Fig. 2H). Interestingly, i.c.v. injection of CREB-HDO significantly blocked the beneficial effects of (*R*)-ketamine (Fig. 2H). Collectively, it is likely that activation of BDNF through CREB phosphorylation in the microglia might play a role in antidepressant-like effects of (*R*)-ketamine.

### Effects of BDNF exon IV-HDO on the antidepressant-like effects of (*R*)-ketamine

**Fig. 3:**
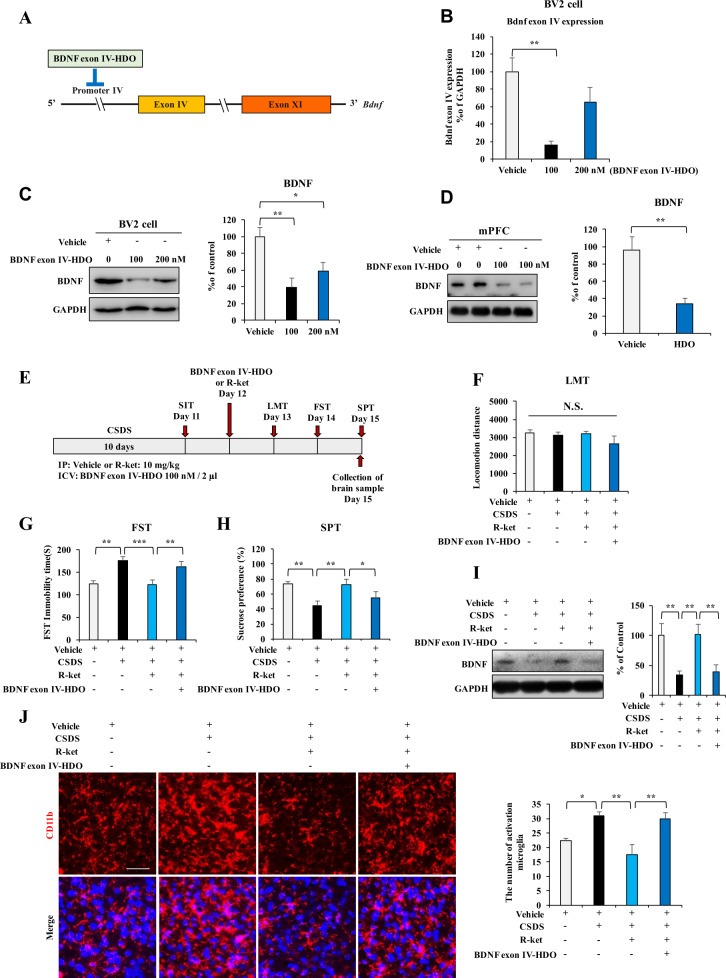
Effects of BDNF exon IV-HDO on antidepressant-like effects of (*R*)-ketamine. **A** Graphical illustration of BDNF exon IV-HDO to inhibit BDNF exon IV promoter expression. **B**, **C** qPCR and western blot assay for BDNF exon IV promoter and BDNF protein expression in the BV2 cells after administration of BDNF exon IV-HDO. The data are the mean ± SEM (*n* = 5). *P < 0.05; **P < 0.01 (one-way ANOVA). **D** Expression of BDNF in the mPFC of adult mice 72 h after i.c.v. administration of BDNF exon IV-HDO. The data are the mean ± SEM (*n* = 4). *P < 0.05 (Student *t* test). **E** The schedule of CSDS, treatment, behavioral tests, and sample collection. CSDS was performed from day 1 to day 10. On day 11, SIT was performed to select CSDS susceptible mice. BDNF exon IV-HDO or vehicle was injected i.c.v. to mice 30 min before i.p. injection of (*R*)-ketamine (10 mg/kg). Subsequently, behavioral tests such as LMT, FST, and SPT were performed. **F** LMT. **G** FST. **H** SPT. The data are the mean ± SEM (*n* = 9). *P < 0.05; **P < 0.01; ***P < 0.001 (one-way ANOVA). **I** Expression of BDNF in the mPFC. The data are the mean ± SEM (*n* = 5). **P < 0.01 (one-way ANOVA). **J** The immunofluorescence analysis for CD11b (for microglia) in the mPFC. The data are the mean ± SEM (*n* = 5). *P < 0.05; **P < 0.01 (one-way ANOVA). Scale bar = 50 μm.

To further identify antidepressant-like effects of (*R*)-ketamine via promoting BDNF expression through activation of CREB in microglia, we designed BDNF exon IV-HDO to silence the binding sequence of CREB (Fig. 3A). Using qPCR and western blot assay, the BDNF exon IV-HDO (100 nM) significantly decreased *Bdnf* exon IV mRNA and BDNF protein expression in the BV2 cells (Fig. 3B, C). In addition, i.c.v. injection of BDNF exon IV-HDO (100 nM) significantly decreased the expression of BDNF in the mPFC of adult mice (Fig. 3D). These results show that BDNF exon IV-HDO can efficiently silence *Bdnf* exon IV mRNA and BDNF protein expression in vitro and in vivo.

Next, we examined whether i.c.v. injection of BDNF exon IV-HDO (100 nM, 2 μl) affects antidepressant-like effects of (*R*)-ketamine in the CSDS susceptible mice (Fig. 3E). Single i.c.v. injection of BDNF exon IV-HDO significantly blocked antidepressant-like effects of (*R*)-ketamine in the CSDS susceptible mice (Fig. 3E–H). There were no changes in locomotion among the four groups (Fig. 3F). In the FST and SPT, BDNF exon IV-HDO significantly blocked antidepressant-like effects of (*R*)-ketamine in the CSDS susceptible mice (Fig. 3G, H).

Western blot analysis revealed that BDNF exon IV-HDO significantly blocked the effects of (*R*)-ketamine on reduced expression of BDNF in the mPFC of CSDS susceptible mice (Fig. 3I). Immunofluorescence staining using CD11b showed that BDNF exon IV-HDO significantly blocked the beneficial effects of (*R*)-ketamine on the increased microglial activation in the mPFC of CSDS susceptible mice (Fig. 3J). These results suggest that activation of BDNF via activation of CREB in microglia might play a role in the antidepressant-like effects of (*R*)-ketamine.

### Effect of microglia depletion on antidepressant-like effects of (*R*)-ketamine

**Fig. 4:**
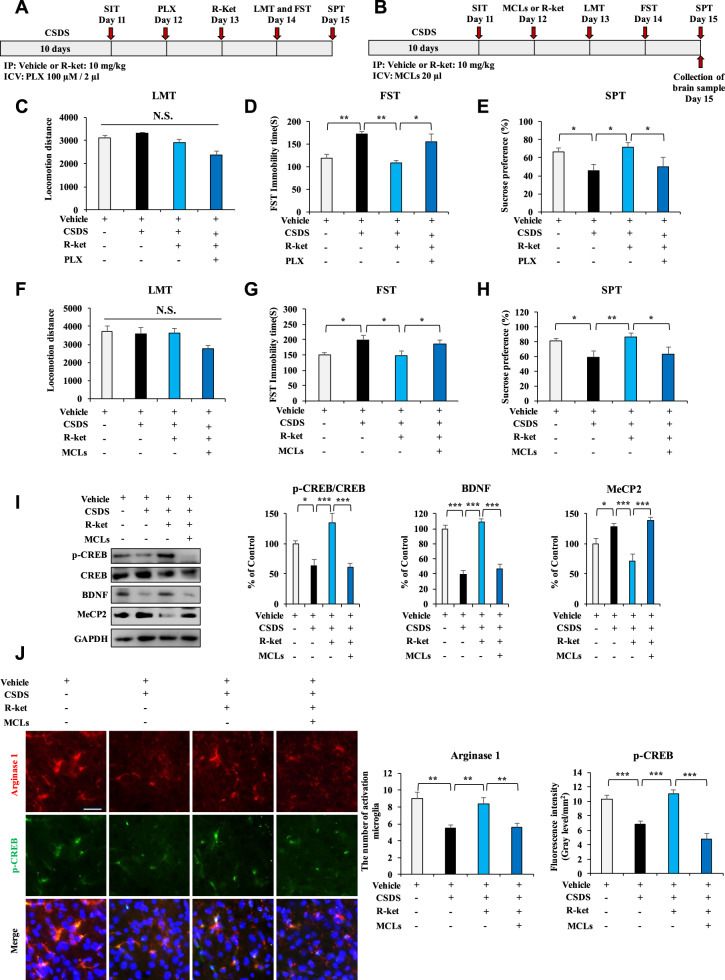
Effects of PLX3397 and MCLs on the antidepressant-like effects of (*R*)-ketamine in the CSDS susceptible mice. **A** The schedule of treatment, behavioral tests, and collection of samples. CSDS was performed from day 1 to day 10. On day 11, SIT was performed to select CSDS susceptible mice. On day 12, PLX3397 or vehicle was injected i.c.v. to CSDS susceptible mice. (*R*)-ketamine (10 mg/kg) or saline was administered i.p. 24 h after PLX injection. Subsequently, behavioral tests such as LMT, FST, and SPT were performed. **B** The schedule of treatment, behavioral tests, and collection of samples. CSDS was performed from day 1 to day 10. On day 11, SIT was performed to select CSDS susceptible mice. On day 12, MCLs or vehicle was injected i.c.v. to CSDS susceptible mice 30 min before i.p. injection of (*R*)-ketamine (10 mg/kg) or saline (10 ml/kg). Subsequently, behavioral tests such as LMT, FST, and SPT were performed. **C**, **F**: LMT. **D**, **G**: FST. **E**, **H**: SPT. The data are the mean ± SEM (*n* = 7 or 8). *P < 0.05; **P < 0.01 (one-way ANOVA). **I** The ratio of p-CREB/CREB, expression of BDNF, and MeCP2 in the mPFC. The data are the mean ± SEM (*n* = 5). *P < 0.05; ***P < 0.001 (one-way ANOVA). **J** The immunofluorescence analysis for arginase1 and p-CREB in the mPFC. The data are the mean ± SEM (*n* = 5). **P < 0.01; ***P < 0.001 (one-way ANOVA). Scale bar = 50 μm.

Activation of CSF1R is essential for survival in microglia [66]. It is reported that i.c.v. injection of PLX3397 (CSF1R inhibitor) reduced the Iba1 protein in the mouse mPFC [64]. To examine the role of microglia in the antidepressant-like effects of (*R*)-ketamine, we performed microglial depletion using PLX3397. In this study, we used the time (24 h) of PLX3397 (100 μM, 2 μl) as previously reported [64]. Microglial depletion by PLX3397 significantly blocked antidepressant-like effects of (*R*)-ketamine in the CSDS susceptible mice (Fig. 4A, C–E). There were no changes in locomotion among the four groups (Fig. 4C). In the FST and SPT, PLX3397 significantly blocked antidepressant-like effects of (*R*)-ketamine in the CSDS susceptible mice (Fig. 4D, E). Collectively, partial depletion of microglia by PLX3397 significantly attenuated antidepressant-like effects of (*R*)-ketamine.

In the CNS, microglial activation is heterogeneous, which can be categorized into two opposite phenotypes: pro-inflammatory and anti-inflammatory, depending on the phenotypes. Microglia can produce either cytotoxic or neuroprotective effects [67]. MCLs are anti-inflammatory phenotypes of microglial inhibitor [68]. Single i.c.v. injection of MCLs significantly decreased expression of arginase1 (anti-inflammatory phenotype microglia marker) and BDNF in the mPFC of mice, suggesting that MCLs can efficiently decrease anti-inflammatory phenotype microglia expression (Fig. S9), consistent with the previous report [68]. We examined the effects of MCLs on the antidepressant-like effects of (*R*)-ketamine in CSDS model (Fig. 4B). Single i.c.v. injection of MCLs significantly blocked antidepressant-like effects of (*R*)-ketamine in the CSDS susceptible mice (Fig. 4F–H). There were no changes in locomotion among the four groups (Fig. 4F). In the FST and SPT, MCLs significantly blocked antidepressant-like effects of (*R*)-ketamine in the CSDS susceptible mice (Fig. 4G, H). Western blot analysis showed that MCLs significantly blocked the beneficial effects of (*R*)-ketamine for p-CREB/CREB ratio, expression of BDNF and MeCP2 in the mPFC of CSDS susceptible mice (Fig. 4I). Furthermore, immunofluorescence staining showed that arginase1 and p-CREB had co-localization in the mPFC (Fig. 4J). CSDS susceptible mice showed the decreased expression of arginase1 and p-CREB in the mPFC, and (*R*)-ketamine significantly ameliorated the decreased expression of arginase1 and p-CREB in the mPFC of CSDS susceptible mice (Fig. 4J). Interestingly, the beneficial effects of (*R*)-ketamine were significantly blocked by MCLs (Fig. 4J). The results suggest that activation of BDNF expression in anti-inflammatory phenotype of microglia might play a role in the antidepressant-like effects of (*R*)-ketamine.

### Role of synaptic plasticity in the antidepressant-like effects of (*R*)-ketamine

**Fig. 5:**
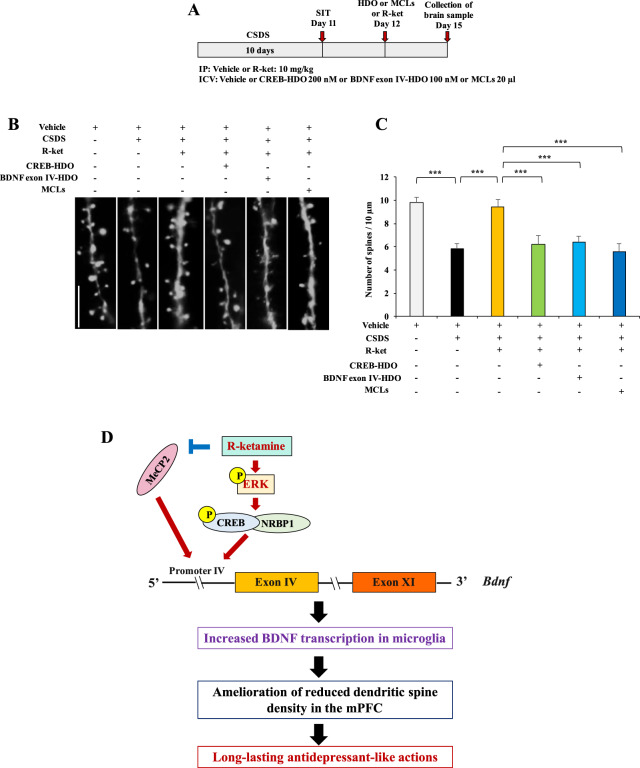
Effects of CREB-HDO, BDNF exon IV-HDO, or MCLs on beneficial effects of (*R*)-ketamine for reduced dendritic spine density in the mPFC of CSDS susceptible mice. **A** The schedule of CSDS, treatment, and collection of samples. CSDS of Thy1-YFP mice was performed from day 1 to day 10. On day 11, SIT was performed to select CSDS susceptible mice. On day 12, HDO, MCLs or vehicle was injected i.c.v. to CSDS susceptible mice 30 min before i.p. injection of (*R*)-ketamine (10 mg/kg) or saline (10 ml/kg). On day 15, brain samples were collected. **B** The representative photomicrographs for dendritic spine density in the mPFC. Scale bar = 10 μm. **C** The quantification analysis of dendritic spine density in the mPFC. The data are the mean ± SEM (*n* = 5). ***P < 0.001 (one-way ANOVA). **D** Working model for the longer-lasting antidepressant-like effects of (*R*)-ketamine. (*R*)-ketamine produces long-lasting antidepressant-like effects through microglial NRBP1-CREB-MeCP2-BDNF signaling in the mPFC.

Using Thy1-YFP mice, we found that the dendritic spine density in the mPFC of CSDS susceptible mice was significantly lower than control mice, and that (*R*)-ketamine significantly ameliorated the reduced dendritic spine density in the mPFC of CSDS susceptible mice (Fig. 5A–C). The beneficial effects of (*R*)-ketamine on the dendritic spine density in the mPFC from CSDS susceptible mice were significantly blocked by single i.c.v. injection of CREB-HDO, BDNF exon IV-HDO or MCLs (Fig. 5A–C). The results suggest that synaptogenesis via CREB-BDNF signaling in microglia might play a role in the antidepressant-like effects of (*R*)-ketamine.

## Discussion

Using iTRAQ, we identified NRBP1 as potential target for long-lasting antidepressant-like effects of (*R*)-ketamine compared with (*S*)-ketamine. We found that NRBP1 is localized in microglia of mPFC of adult mouse brain. Furthermore, NRBP1 binds to CREB in BV2 cells. Moreover, (*R*)-ketamine increased expression of NRBP1, BDNF and ratio of p-CREB/CREB through ERK activation in primary microglia. A recent study showed the role of BDNF-TrkB signaling via ERK in hippocampal CA1 for antidepressant-like action of (*R,S*)-ketamine [69]. Our study suggests that (*R*)-ketamine can enhance BDNF expression through ERK-NRBP1-CREB signaling in microglia.

Luciferase assays showed that both (*R*)-ketamine and (*S*)-ketamine could activate *Bdnf* exon IV promoter via CREB phosphorylation. Importantly, (*R*)-ketamine was more potent than (*S*)-ketamine. Given the affinity of two enantiomers at NMDAR [25], it is unlikely that NMDAR plays a major role in the activation of *Bdnf* exon IV promotor by (*R*)-ketamine. Furthermore, we found that (*R*)-ketamine produced antidepressant-like effects in CSDS susceptible mice, and that (*R*)-ketamine improved both decreased CD11b immunoreactivity and increased p-CREB immunoreactivity in the mPFC of CSDS susceptible mice. Moreover, single i.c.v. injection of CREB-HDO or BDNF exon IV-HDO blocked antidepressant-like effects of (*R*)-ketamine in CSDS susceptible mice. Collectively, activation of BDNF transcription via NRBP1-CREB in microglia by (*R*)-ketamine may play a role in its antidepressant-like actions (Fig. 5D).

Microglia are the only cell type that express CSF1R. CSF1R knockout mice are devoid of microglia. In this study, we used i.c.v. injection of PLX3397 to delete microglia in the brain because chronic systemic administration of CSF1R inhibitor caused abnormal composition of gut microbiota which might be involved in behavioral abnormalities [70]. We found that partial depletion of microglia in the mPFC by PLX3397 blocked antidepressant-like effects of (*R*)-ketamine in CSDS susceptible mice, consistent with recent report [64]. Furthermore, i.c.v. injection of MCLs blocked beneficial effects of (*R*)-ketamine on depression-like phenotypes, reduced ratio of p-CREB/CREB, reduced expression of BDNF and increased expression of MeCP2 in the mPFC of CSDS susceptible mice. Thus, it appears likely that microglial CREB-BDNF signaling in the mPFC might contribute to antidepressant-like effects of (*R*)-ketamine. A recent meta-analysis using positron emission tomography data showed increased expression of microglia in the brain of patients with depression [71].

MeCP2 is a transcriptional repressor identified as the protein that binds to methylated CpG sites [72]. In this study, we found that (*R*)-ketamine showed antidepressant-like effects in CSDS susceptible mice by activating BDNF as well as by inhibiting MeCP2, similar to the recent report [53]. In contrast, a recent study showed that phosphorylation of MeCP2 is essential for the sustained antidepressant-like effects of (*R,S*)-ketamine in mice [73]. However, they used *Mecp2* knock-in mice and *Bdnf* conditional knockout mice without depression-like phenotype [73]. Further study using these mice with depression-like phenotypes is needed [24]. Since we did not examine phosphorylation of MeCP2 in our samples, it is interesting to examine the role of phosphorylation of MeCP2 in the antidepressant-like effects of (*R*)-ketamine.

It is suggested that reduced dendritic spine density in the mPFC of CSDS susceptible mice might be involved in depression-like behaviors [74, 75]. In this study, we found that single i.c.v. injection of CREB-HDO, BDNF exon IV-HDO, or MCLs blocked the beneficial effects of (*R*)-ketamine on the reduced dendritic spine density in the mPFC of CSDS susceptible mice. Overall, it is likely that (*R*)-ketamine can exert antidepressant-like effects by normalizing reduced dendritic spine density in the mPFC of CSDS susceptible mice.

This article has some limitations. First, we used CSDS model as animal model of depression. The advantages and limitations of animal models such as CSDS for translation in humans were pointed [76]. Second, we selected mPFC of mouse brain since mPFC is implicated in depression-like phenotypes [27, 47, 48]. In contrast, other brain regions such as hippocampus and nucleus accumbens are known to play a role in depression-like phenotypes [27, 41, 48, 50, 77, 78]. Further study using other brain regions in the antidepressant-like effects of (*R*)-ketamine is important. Third, we focused on microglia in this study. However, further study using other cell types (i.e., astrocyte, neuron) is also needed. Finally, we examined depression-like phenotypes in this study. A new study showed that ERK phosphorylation in the amygdala was associated with anxiety symptoms [79]. Therefore, it is of interest to investigate the effects of (*R*)-ketamine in anxiety.

In conclusion, the current data show that ERK-NRBP1-CREB-BDNF signaling in microglia might contribute to antidepressant-like effects of (*R*)-ketamine compared to (*S*)-ketamine. Therefore, it is likely that ERK-NRBP1-CREB-BDNF signaling in microglia would be a new target for depression.

## Supplementary information

Supplemental information
