# Correction: Microglial ERK-NRBP1-CREB-BDNF signaling in sustained antidepressant actions of (*R*)-ketamine

**DOI:** 10.1038/s41380-021-01417-2

**Published:** 2021-12-08

**Authors:** Wei Yao, Qianqian Cao, Shilin Luo, Lujuan He, Chun Yang, Jiaxu Chen, Qi Qi, Kenji Hashimoto, Ji-chun Zhang

**Affiliations:** 1 Guangzhou Key Laboratory of Formula-Pattern Research Center, School of Traditional Chinese Medicine, Jinan University, Guangzhou, China; 2 Department of Physiology, School of Medicine, Jinan University, Guangzhou, China; 3 Department of Pharmacy, The Second Xiangya Hospital, Central South University, Changsha, China; 4 Division of Clinical Neuroscience, Chiba University Center for Forensic Mental Health, Chiba, Japan; 5 MOE Key Laboratory of Tumor Molecular Biology, Clinical Translational Center for Targeted Drug, Department of Pharmacology, School of Medicine, Jinan University, Guangzhou, China; 6 Present Address: Department of Anesthesiology and Perioperative Medicine, The First Affiliated Hospital of Nanjing Medical University, Nanjing, China

**Keywords:** Neuroscience, Depression, Neuroscience, Depression

Correction to: *Molecular Psychiatry* 10.1038/s41380-021-01377-7, published online 24 November 2021

The article “Microglial ERK-NRBP1-CREB-BDNF signaling in sustained antidepressant actions of (*R*)-ketamine,” written by Wei Yao, Qianqian Cao, Shilin Luo, Lujuan He, Chun Yang, Jiaxu Chen, Qi Qi, Kenji Hashimoto, and Ji-chun Zhang, was originally published electronically on the publisher’s internet portal on November 24, 2021, without open access. With the author(s)’ decision to opt for Open Choice, the copyright of the article changed on November 28, 2021, to © The Author(s) 2021 and the article is forthwith distributed under a Creative Commons Attribution 4.0 International License, which permits use, sharing, adaptation, distribution, and reproduction in any medium or format, as long as you give appropriate credit to the original author(s) and the source, provide a link to the Creative Commons license, and indicate if changes were made. The images or other third-party material in this article are included in the article’s Creative Commons license, unless indicated otherwise in a credit line to the material. If material is not included in the article’s Creative Commons license and your intended use is not permitted by statutory regulation or exceeds the permitted use, you will need to obtain permission directly from the copyright holder. To view a copy of this license, visit http://creativecommons.org/licenses/by/4.0.

