# Supplementary material for: Microglial ERK-NRBP1-CREB-BDNF signaling in sustained antidepressant actions of (R)-ketamine
Source: Mol Psychiatry. 2021 Nov 24;27(3):1618–29. doi: 10.1038/s41380-021-01377-7 (PMC9095473; doi:10.1038/s41380-021-01377-7)
Supplement: Supplementary file 1 — Supplemental information [file 41380_2021_1377_MOESM1_ESM.docx]

**Supplemental information**

**Microglial ERK-NRBP1-CREB-BDNF signaling in sustained antidepressant actions of (*R*)-ketamine**

Wei Yao^1^**^#^**, Qianqian Cao^2^**^#^**, Shilin Luo^3^**^#^**, Lujuan He^2^, Chun Yang^4^, Jiaxu Chen^1^, Qi Qi^5*^, Kenji Hashimoto^4*^, and Ji-chun Zhang^2*^

^1^Guangzhou Key Laboratory of Formula-pattern Research Center, School of Traditional Chinese Medicine, Jinan University, Guangzhou 510632, China; ^2^Department of Physiology, School of Medicine, Jinan University, Guangzhou, 510632, China; ^3^Department of Pharmacy, the Second Xiangya Hospital, Central South University, Changsha 410011, China; ^4^Division of Clinical Neuroscience, Chiba University Center for Forensic Mental Health, Chiba 260–8670, Japan; and ^5^MOE Key Laboratory of Tumor Molecular Biology, Clinical Translational Center for Targeted Drug, Department of Pharmacology, School of Medicine, Jinan University, Guangzhou 510632, China.

**Correspondence:** Ji-chun Zhang ([jczhang@jnu.edu.cn)](mailto:jczhang@jnu.edu.cn)), Kenji Hashimoto ([hashimoto@faculty.chiba-u.jp)](mailto:hashimoto@faculty.chiba-u.jp)) and Qi Qi ([[qiqikc@jnu.edu.cn](mailto:qiqikc@jnu.edu.cn))](mailto:qiqikc@jnu.edu.cn))

**^#^**These authors contributed equally to this work.

Present address of Dr. Chun Yang is at Department of Anesthesiology and Perioperative Medicine, The First Affiliated Hospital of Nanjing Medical University, Nanjing, China.

**Methods and Materials**

**Animals**

Male adult C57BL/6 mice, aged 8 weeks (body weight 20-25g, Japan SLC, Inc., Hamamatsu, Japan), male CD1 mice, aged 14 weeks (body weight 40-45g, Japan SLC, Inc., Hamamatsu, Japan) were used in the experiments for iTRAQ-based proteomics assay. Animals were housed under controlled temperature and 12 h light/dark cycles (lights on between 07:00-19:00), with *ad libitum* food and water. The study was approved by the Chiba University Institutional Animal Care and Use Committee. The male adult C57BL/6 mice (8 weeks old, 20–25 g each, Guangdong Experimental Animal Center, China), CD1 mice (14 weeks old, 40–45 g each, Guangdong Experimental Animal Center, China), and male adult Thy1-Yellow fluorescent protein (YFP) mice (1) were used. The animals were housed under controlled temperature and kept on a 12-h light/dark cycle (lights on between 07:00 and 19:00), with *ad libitum* access to food and water. The protocol was approved by the Jinan University Institutional Animal Care and Use Committee. All experiments were carried out following the Guide for Animal Experimentation of Jinan University. The animals in this study were randomly allocated to experimental groups with age-matched. The sample sizes were based on the previous experience with the experimental design (3,4,7-10). Since several batched of mice were tested independently, we used pooled together for final analyses. Therefore, the group sizes are not exactly the same. The criteria were not pre-established.

**Compounds, HDO, siRNA and Plasmids**

(*R*)-ketamine hydrochloride and (*S*)-ketamine hydrochloride were prepared by recrystallization of (*R,S*)-ketamine and D-(-)-tartaric acid (or L- (+)-tartaric acid), respectively (2). (2*R*,6*R*)-hydroxynorketamine [(2*R*,6*R*)-HNK] (SML1873, Sigma-Aldrich, St. Louis, MO, USA) was dissolved in the saline. Lipopolysaccharide (LPS: L-4130, serotype 0111: B4, Sigma-Aldrich, St. Louis, MO, USA) was dissolved in physiological saline. SL327 (an ERK inhibitor; MedChemExpress, NJ, USA) was dissolved in dimethyl sulfoxide (DMSO). PLX3397 [colony-stimulating factor 1 receptor (CSF1R) inhibitor, Bioactive Compounds Expert, USA] was dissolved in 10% DMSO and 90% sulfobutylether-β-cyclodextrin (SBE-β-CD). Mannosylated clodronate liposomes (MCLs) (Encapsula Nano Science, TN, USA) were applied to polarized microglia *in vivo* to assess specificity in promoting anti-inflammatory phenotype of microglia apoptosis. The doses of (*R*)-ketamine, (*S*)-ketamine, LPS, SL327, PLX3397, and MCLs were selected as reported previously (2-11).

The antisense oligonucleotides (ASO) and cRNA targeting CREB and BDNF exon IV were synthesized from TsingKe Biological Technology (Wuhan, China) and solubilized in 0.9% sterile saline immediately before use. For the generation of CREB-DNA/RNA heteroduplex oligonucleotides (HDO) or BDNF exon IV-HDO, equimolar amounts of DNA and cRNA strands were heated in 0.9% sterile saline at 95 °C for 5 min and slowly cooled to room temperature. CREB-HDO and BDNF exon IV-HDO that carried locked nucleic acids (LNA) at each end flanking the central base of DNA and with or without CY5 label, and carried 2’-O-methl at each end flanking the central base of cRNA. The sequences of ASOs and cRNA targeting CREB and BDNF exon IV used in our experiments were listed below. CREB-ASO: T(L)^G(L)^G^T^C^A^T^C^T^A^G^T^C^A^C^C^G^G(L)^T(L)^G(L); CREB-cRNA: c(M)^a(M)^c(M)^cggugacuagaugac^c(M)^a(M). BDNF exon IV-ASO: C(L)^A(L)^G^T^C^A^C^T^A^C^T^T^G^T^C^A^A^A^G(L)^T(L)^A(L); BDNF exon IV-cRNA: u(M)^a(M)^c(M)^uuugacaaguagugac^u(M)^g(M). L is locked nucleic acids. M is 2’-O-methyl modifications. ^ is phosphorothioate bond. Other reagents were purchased commercially. The sequences of CREB-ASO and BDNF exon IV-ASO were selected as reported previously (12,13).

The siRNA-NRBP1 (GTCGAGAAGAGCAGAAGAA) was synthesized by TsingKe Biological Technology (Wuhan, China). The sequences of siRNA-NRBP1 was selected as reported previously (14). The siRNA-CREB (6590s) was purchased from Cell Signaling Technology. The BDNF exon IV luciferase reporter plasmid and CREB motif mutated BDNF exon IV luciferase reporter plasmid was synthesized by TsingKe Biological Technology (Wuhan, China).

**Cell cultures**

HEK293T cells and BV2 cells (for microglia) were cultured in high-glucose DMEM supplemented with 10% fetal bovine serum (FBS) (Excell Bio.) and penicillin (100 units/mL)–streptomycin (100 μg/mL) (PS) (all from Hyclone). Cells were incubated at 37°C in a humidified incubator containing 5% CO_2_. HEK293T and BV2 cells were a gift from Dr. Zhentao Zhang (Department of Neurology, Renmin Hospital of Wuhan University, Wuhan, China). All the cell lines were tested for mycoplasma contamination.

For the primary culture of microglia, the glia was prepared from newborn C57BL/6 mice within 24 h. The whole brains were taken after disinfection with 75% alcohol. The whole brains were placed in the 4°C precooled sterile D-Hank’s buffer, slightly cleaned the blood cells, and then transferred to the petri dish containing the precooled D-Hank’s buffer. The meninges and blood vessels on the brain surface were stripped under the microscope, and the brain tissue was transferred to the ice centrifuge tube. 0.25% trypsin was added for digestion at room temperature for 10 min, and the digestion was stopped with complete medium DMEM/F-12. After the brain tissue was fully blown away, the liquid in the centrifuge tube was passed through a 100 μm screen, the filtrate was centrifuged at room temperature for 1500 RPM for 10 min, and the supernatant was absorbed and discarded, and the cells were resuspended with DMEM/F-12 medium. After thoroughly blowing, inoculation was carried out at a density of 3 ×10^6^ /mL into the T75 culture flask coated with cell adhesive and placed in an incubator at 37°C and 5% CO_2_ for culture. The liquid was changed every 3 days. After about 10 ~ 12 days of culture, 0.25% trypsin diluted in DMEM at a dilution ratio of 1:4 was used to dissociate astrocytes for 30 minutes. After removal of astrocytes, microglia were planted in culture plates for use (15).

**Chronic social defeat stress (CSDS)**

For the CSDS depression model, the C57BL/6 mice or Thy1-YFP mice were exposed for different CD1 mice for 10 min for 10 days according to the previous methods (6,7,16). The CD1 mouse and C57BL/6 mice or Thy1-YFP mice were housed in the half of the cage that separated by using perforated Plexiglas divider, which can allow visual, olfactory, and auditory contact in the 24 hours after social defeat session. After 10 days social defeat session, the C57BL/6 mice or Thy1-YFP mice were raised separately. The susceptible and unsusceptible mice were identified by social interaction test.

In the social interaction test, an open box (42 × 42 cm) was used, which has interaction zone including a mesh-plastic target box (10 × 4.5 cm) and two opposing corner zones. This test is divided into two parts (no social target and social target). For the no social target, the test mouse was placed into open field arena for 2.5 min with no social target (no CD1 mouse) in the mesh-plastic target box. After no social target test, mouse was placed into open field arena again in second 2.5 min with social target (a novel CD1 mouse) in the mesh-plastic target box. The residence time in the interaction zone was counted by using Ethovision XT 14.0 software (Noldus), the time of ratio for social target and no social target was calculated. About 70% of mice were susceptible after social defeat stress (6,7,16).

**Intracerebroventricular (i.c.v.) injection, and behavioral tests**

Mice were anesthetized with isoflurane and fixed to the stereotaxic apparatus. CREB-HDO (200 nM in 2 μl), BDNF exon IV-HDO (100 nM in 2 μl), PLX3397 (100 μM in 2 μl) or MCLs (20 µl) were injected into the right lateral ventricle following the stereotaxic coordinates: 0.8 mm lateral, 2.1 mm ventral, and 0.74 mm from Bregma. The needle remained in place for 5 minutes after injection of the drugs, followed by slow removal. The mice were placed on a heating pad until recovery from anesthesia.

Behavioral tests including locomotion, forced swimming test (FST), and 1% sucrose preference test (SPT), as previously reported (5-8). Locomotion: the locomotor activities of mice were analyzed by using Ethovision XT 14.0 software (Noldus). The cumulative exercise was recorded in 60 minutes. FST: The mice were placed individually in a cylinder (diameter: 23 cm; height: 31 cm) containing 15 cm of water, maintained at 23 ± 1 °C. Mice were monitored using a video tracking system (Ethovision XT 14.0) for 6 minutes. SPT: The mice were habituated to a 1% sucrose solution for 48 h before the test day. And then the mice were deprived of water and food for 4 h, followed by a preference test with water and 1% sucrose for 1 h. The bottles containing water and sucrose were weighed before and at the end of this period and the sucrose preference (%) was determined (5-8).

**Immunoprecipitation**

The cells sample were lysed in lysis buffer and centrifuged for 15 min at 16,000 g. The supernatant was incubated with anti-NRBP1 or anti-IgG antibody and protein A/G-agarose overnight at 4 °C. After extensive washing, the bound proteins were eluted from the beads by boiling in sample buffer and subjected to Western blot analyses. **Quantitative real-time PCR assay**

Levels of *Bdnf* exon IV mRNA were analyzed by quantitative real-time PCR. RNA was extracted by using Eastep® Super Kit (Promega), and then reverse transcription was performed with GoScript^TM^ Reverse Transcriptase Mix, Oligo (dT) (Promega). All real-time PCR reactions were performed by using the 788BR05175 Real-Time PCR System and ChamQ^TM^ SYBR® qPCR Master Mix Kit (Vazyme). The target gene expression was calculated as 2−ΔΔCt method. Forty cycles of PCR amplification were performed as follows: denature at 95°C for 30 s, anneal at 55°C for 30 s, and extend for 30 s at 72°C. The primer sequences were: *Bdnf* exon IV forward 5’ GGCTTCTGTGTGCGTGAATTTGC 3’; reverse 5’ AAAGTGGGTGGGAGTCCACGAG3’ based on previously published results (17)

**Western blotting assay**

Cells or brain samples were lysed in RIPA buffer (20 mM pH 7.5 Tris-HCl, 150 mM NaCl, 1 mM Na_2_EDTA, 1 mM EGTA, 1% Triton, 2.5 mM sodium pyrophosphate, 1 mM beta-glycerophosphate, 1 mM Na_3_VO_4_, 1 μg/ml leupeptin, 1 mM phenylmethylsulfonyl fluoride). The concentrations of total proteins were examined by Bradford assay. 30 μg Proteins were resolved on 7.5%, 10%, or 15% polyacrylamide gels, according to each marker’s molecular weight, and then transferred to polyvinylidenedifluoride (PVDF) membrane. For the immunodetection, the blots were blocked with 2% BSA plus 5% nonfat dry milk in TBST (TBS + 0.1% Tween-20) for 1 h at room temperature (RT), and then incubated with primary antibodies (The concentration is selected with the manufacturer’s instructions) overnight at 4°C. Next day, blots were washed three times in TBST and incubated with horseradish peroxidase conjugated anti-rabbit antibody (1:5000) or anti-mouse antibody (1:5000) for 1hour, at RT. After the three times washes with TBST, the bands were detected by using enhanced chemiluminescence (ECL) detection reagents (GE Healthcare) and exposed to Tanon-5200CE imaging system (Tanon, Shanghai, China). The quantification was carried out with ImageJ software. The primary antibodies used in assays were listed below: NRBP1 antibody (A10301) was purchased from ABclomal, phospho-ERK antibody (4695), ERK antibody (4377), phospho-CREB antibody (9198S), CREB antibody (9197S) and arginase1 (93668S) was purchased from Cell Signaling Technology, MeCP2 antibody (M6818) was purchased from Sigma. BDNF antibody (ab108319) was purchased from abcam, GAPDH antibody was purchased from EarthOx. The HRP-conjugated anti-rabbit IgG antibody, and anti-mouse IgG antibody were purchased from BIO-RAD.

**Luciferase assay**

Cells in 6-wells or 12-wells plates were transfected with BDNF exon IV luciferase reporter together, pRL-TK Renilla luciferase plasmid (Promega), and different kinds of plasmids or siRNA. Following transfection for 24 h, the cells were collected and subjected the dual-luciferase reporter assay kit (Promega) according to the manual.

**Chromatin immunoprecipitation (ChIP) assay**

After treatment with vehicle or (*R*)-ketamine or (*S*)-ketamine, the cells were subjected to the ChIP assay protocol according to manual of the SimpleChIP^®^ Enzymatic Chromatin IP Kit (Cell Signaling). For the p-CREB antibody, 7.5 μg of p-CREB antibody (9198S, Cell Signaling Technology) was added to the homogenate for the sample. For the PCR analysis. Specific primers were for the amplification of promoter region of *Bdnf* exon IV, which contains a putative CREB binding site. The primer sequences were: forward 5’ GGCTTCTGTGTGCGTGAATTTGC 3’; reverse 5’ AAAGTGGGTGGGAGTCCACGAG 3’ based on previously published results (17). The PCR amplicon was separated with a 2% agarose gel after 35 cycles of PCR (denature at 95°C for 30 s, anneal at 58°C for 30 s, and extend for 30 s at 72°C).

**Immunofluorescence staining**

The mice were anesthetized with sodium pentobarbital and perfused transcardially with 10 ml of isotonic saline, followed by 40 ml of ice-cold 4% paraformaldehyde in 0.1-M phosphate buffer (pH 7.4). After the perfused the brain samples were collected and postfixed overnight at 4°C. In the next day, 50-μm thick serial coronal sections of brain tissue were cut in ice-cold, 0.01-M phosphate-buffered saline (pH 7.5), using a vibrating blade microtome (VT1000S, Leica Microsystems AG, Wetzlar, Germany). Brain sections were identified according the previously reported (7). For the immunofluorescence staining, the slides with cells were fixed by 4% paraformaldehyde. And then the slides with cells or mice brain sections were incubated with 3% hydrogen peroxide at room temperature for 10 minutes followed by blocking and incubation with primary antibodies (anti-phospho-CREB antibody (1:500, 9198S, Cell Signaling Technology), anti-CD11b (1:500, 2151423, Invitrogen) and anti-arginase1(1:500, 93668S, Cell Signaling Technology), anti-CamK2α (1:500, 50049S, Cell Signaling Technology), anti-GABA (1:500, A0310, Sigma-Aldrich)) for 48 h at 4°C. On the third day, the slides with cells or brain section were incubated with an Alexa Fluor 488 or 568 conjugated isotype-specific secondary antibody for 1 h at room temperature. Images were then collected with an Olympus confocal microscope. The fluorescence intensity was quantified using Image J analysis software.

**Dendritic spine analysis**

CSDS of Thy1-YFP mice was performed for 10 days (day 1 – day 10). After the social interaction test on day 11, CSDS susceptible mice were selected. On day 12, HDO, MCLs or vehicle was injected i.c.v. to CSDS susceptible mice 30 min before i.p. injection of (*R*)-ketamine (10 mg/kg) or saline (10 ml/kg). Three days after administration of (*R*)-ketamine or saline, all mice were deeply anesthetized with sodium pentobarbital and perfused transcardially with 10 ml of isotonic saline, followed by 40 ml of ice-cold 4% paraformaldehyde in 0.1-M phosphate buffer (pH 7.4). Brains were removed from the skulls and postfixed overnight at 4°C with the same fixative. For dendritic spine analysis, 50-μm thick serial coronal sections of brain tissue were cut in ice-cold, 0.01-M phosphate-buffered saline (pH 7.5) using a vibrating blade microtome (VT1000S, Leica Microsystems AG, Wetzlar, Germany). The sections were mounted on gelatinized slides, dehydrated, cleared, and coverslipped under Permount^®^ (Fisher Scientific, Fair Lawn, NJ, USA). Next, sections were observed in a fluorescent microscope (Olympus BX53, Japan), and pictures were then taken and dendritic spine was quantified in 10 μm of each dendritic in a blinded manner.

**References**

1. Feng G, Mellor RH, Bernstein M, Keller-Peck C, Nguyen QT, Wallace M, et al. Imaging neuronal subsets in transgenic mice expressing multiple spectral variants of GFP. Neuron 2000; 28: 41–51.
2. Zhang JC, Li SX, Hashimoto K. [*R*(-)-ketamine shows greater potency and longer lasting antidepressant effects than *S*(+)-ketamine.](http://www.ncbi.nlm.nih.gov/pubmed/24316345) Pharmacol. Biochem. Behav. 2014; 116: 137–141.
3. Yang C, Shirayama Y, Zhang JC, Ren Q, Yao W, Ma M, et al. [*R*-ketamine: a rapid-onset and sustained antidepressant without psychotomimetic side effects.](http://www.ncbi.nlm.nih.gov/pubmed/26327690) Transl Psychiatry 2015; 5: e632.
4. Yang C, Ren Q, Qu Y, Zhang JC, Ma M, Dong C, et al. Mechanistic target of rapamycin-independent antidepressant effects of (*R*)-ketamine in a social defeat stress model. Biol. Psychiatry 2018; 83: 18–28.
5. Zhang JC, Wu J, Fujita Y, Yao W, Ren Q, Yang C, et al. Antidepressant effects of TrkB ligands on depression-like behavior and dendritic changes in mice after inflammation. Int. J. Neuropsychopharmacol. 2014; 18: pyu077.
6. Zhang JC, Yao W, Dong C, Yang C, Ren Q, Ma M, et al. Comparison of ketamine, 7,8-dihydroxyflavone, and ANA-12 antidepressant effects in the social defeat stress model of depression. Psychopharmacology (Berl) 2015; 232: 4325–4335.
7. Zhang JC, Yao W, Dong C, Yang C, Ren Q, Ma M, et al. Blockade of interleukin-6 receptor in the periphery promotes rapid and sustained antidepressant actions: a possible role of gut-microbiota-brain axis. Transl. Psychiatry 2017; 7: e1138.
8. Zhang JC, Yao W, Dong C, Yang C, Ren Q, Ma M, et al. Prophylactic effects of sulforaphane on depression-like behavior and dendritic changes in mice after inflammation. J. Nutri. Biochem. 2017; 39: 134–144.
9. Zhang K, Yang C, Chang L, Sakamoto A, Suzuki T, Fujita Y, et al*.* Essential role of microglial transforming growth factor-β1 in antidepressant actions of (*R*)-ketamine and the novel antidepressant TGF-β1. Transl. Psychiatry 2020; 10: 32.
10. Zhang J, Ma L, Chang L, Pu Y, Qu Y, Hashimoto K. A key role of the subdiaphragmatic vagus nerve in the depression-like phenotype and abnormal composition of gut microbiota in mice after lipopolysaccharide administration. Transl. Psychiatry 2020; 10: 186.
11. Miron VE, Boyd A, Zhao JW, Yuen TJ, Ruckh JM, Shadrach JL, et al. M2 microglia and macrophages drive oligodendrocyte differentiation during CNS remyelination. Nat. Neurosci. 2013; 16: 1211–1218.
12. Gu X, Bo J, Zhang W, Sun X, Zhang J, Yang Y, et al. Intrathecal administration of cyclic AMP response element-binding protein-antisense oligonucleotide attenuates neuropathic pain after peripheral nerve injury and decreases the expression of N-methyl-D-aspartic receptors in mice. Oncol Rep. 2013; 30(1):391-398.
13. Bambah-Mukku D, Travaglia A, Chen DY, Pollonini G, Alberini CM. [A positive autoregulatory BDNF feedback loop via C/EBPβ mediates hippocampal memory consolidation.](https://pubmed.ncbi.nlm.nih.gov/25209292/) J Neurosci. 2014; 34(37):12547-59.
14. Wu Q, Zhou X, Li P, Wang W, Wang J, Tan M, et al. High NRBP1 expression promotes proliferation and correlates with poor prognosis in bladder cancer. J Cancer. 2019 Jul 10;10(18):4270-4277.
15. Yin J, Liu X, He Q, Zhou L, Yuan Z, Zhao S. [Vps35-dependent recycling of Trem2 regulates microglial function.](https://pubmed.ncbi.nlm.nih.gov/27717139/) Traffic. 2016;17(12):1286-1296.
16. Golden SA, Covington HE, 3rd, Berton O, Russo SJ. A standardized protocol for repeated social defeat stress in mice. Nat. Protocols 2011; 6: 1183–1191.
17. Martinowich K, Hattori D, Wu H, Fouse S, He F, Hu Y, et al. DNA methylation-related chromatin remodeling in activity-dependent BDNF gene regulation. Science 2003; 302: 890–893.

**Table S1. The top 10 candidates by iTRAQ analysis**

The iTRAQ analysis of mPFC samples 7 days after a single administration of (*R*)-ketamine (10 mg/kg) or (*S*)-ketamine (10 mg/kg) in the CSDS susceptible mice was performed. Table S1 showed the top 10 proteins which were different between two enantiomers.

**Figure S1. Expression of NRBP1 in the cell types in the mPFC** **of adult mice**

The immunofluorescence staining for NRBP1, Camk2α (for neuron), GABA (for neuron), GFAP (for astrocyte), CD11b (for microglia) in the mPFC of adult mouse. Scale bar = 50 μm.

**Figure S2. Binding of NRBP1 and p-CREB under physiological function and after (*R*)-ketamine or (*S*)-ketamine treatment**

The BV2 cells were treated with vehicle, (*R*)-ketamine (10 μM) or (S)-ketamine (10 μM) for 24 hours. The binding between NRBP1 and p-CREB was confirmed using immunoprecipitation.

**Figure S3. CREB activation through NRBP1 and ERK by (*R*)-ketamine**

**A**: Expression of NRBP1 and the ratio of p-CREB/CREB in BV2 cells treated with siRNA-NRBP1. The data are the mean ± SEM (n = 4). ***P* < 0.01, ****P* < 0.001, **^$^***P* < 0.05, **^$$^***P* < 0.01, **^$$$^***P* < 0.001 (one-way ANOVA). **B**: Expression of NRBP1 and the ratio of p-CREB/CREB in the primary microglia by (*R*)-ketamine or (*S*)-ketamine. The data are the mean ± SEM (n = 4). ***P* < 0.01, ****P* < 0.001, **^$^***P* < 0.05, **^$$$^***P* < 0.001 (one-way ANOVA). **C**: Expression the ratio of p-ERK/ERK, NRBP1, the ratio of p-CREB/CREB and BDNF in the primary microglia by (*R*)-ketamine with or without SL327 (ERK inhibitor). The data are the mean ± SEM (n = 6). ***P* < 0.01, ****P* < 0.001, **^$^***P* < 0.05, **^$$^***P* < 0.01, **^$$$^***P* < 0.001 (one-way ANOVA).

**Figure S4. Effects of (*R*)-ketamine and (2*R,*6*R*)-hydroxynorketamine (HNK) on the BDNF exon IV activation**

The luciferase assay for BDNF exon IV promoter. BDNF exon IV promoter activity in the HEK293T cells treated with (2*R,*6*R*)-HNK (0.1, 1.0, and 10 μM) or (*R*)-ketamine (0.1, 1.0, and 10 μM). The data are the mean ± SEM (n = 4). **P* < 0.05, ****P* < 0.001 compared to vehicle group. **^$^***P* < 0.05, **^$$$^***P* < 0.001 compared to (2*R,*6*R*)-HNK group (one-way ANOVA).

**Figure S5. (*R*)-ketamine or (*S*)-ketamine causes the redistribution of p-CREB and MeCP2 in BV2 cells**

The immunofluorescence for p-CREB and MeCP2 in LPS (1 μg/ml) and/or (*R*)-ketamine (10 μM) and/or (*S*)-ketamine (10 μM)-treated BV2 cells. Scale bar = 50 μm.

**Figure S6. Role of CREB in the beneficial effects of (*R*)-ketamine on reduced expression of p-CREB and BDNF, and increased expression of MeCP2 in the LPS treated-BV2 cells**

The western blot analysis for p-CREB, CREB, BDNF, and MeCP2 expression in LPS (1 μg/ml)-treated BV2 cells with (*R*)-ketamine (10 μM) or (*S*)-ketamine (10 μM). The data are the mean ± SEM (n = 4). *P < 0.05, **P < 0.01 (one-way ANOVA).

**Figure S7. Effects of (*R*)-ketamine on depression-like phenotypes and abnormalities in the expression of NRBP1, p-CREB/CREB, BDNF, and MeCP2 in the CSDS susceptible mice**

**A:** The schedule of CSDS, treatment, and behavioral tests including social interaction test (SIT), locomotion test (LMT), forced swimming test (FST), and sucrose preference test (SPT). **B**: LMT. **C**: FST. **D**: SPT. The data are the mean ± SEM (n = 8 or 9). **P* < 0.05 (one-way ANOVA). **E**: Expression of NRBP1, the ratio of p-CREB/CREB, BDNF, and MeCP2 in the mPFC. The data are the mean ± SEM (n = 5). **P* < 0.05, ***P* < 0.01 (one-way ANOVA). **F**: The immunofluorescence analysis for p-CREB in the mPFC. The data are the mean ± SEM (n = 5). ***P* < 0.01 (one-way ANOVA). Scale bar = 50 μm.

**Figure S8. Effects of CREB-HDO on the expression of CREB and BDNF in the BV2 cells and mPFC of mouse brain**

**A:** Treatment with CREB-HDO (100, 200, or 400 nM) significantly decreased the expressions of CREB and BDNF in the BV2 cells. Mean ± SEM (n = 4). **P < 0.01, ***P < 0.001 (one-way ANOVA). **B**: Single i.c.v. injection of CREB-HDO (200 nM, 2 μl) significantly decreased the expressions of CREB and BDNF in the mPFC of mouse brain. The data are the mean ± SEM (n = 4). ***P < 0.001 (Student t-test).

**Figure S9. ICV injection of MCLs decreased the expression of arginase1 and BDNF in the mPFC**

MCLs decreased arginase1 and BDNF expression in the mPFC of adult mice. The data are the mean ± SEM (n = 4). ****P* < 0.001 (Student’s t-test).
